# High-fat meal effect on LDL, HDL, and VLDL particle size and number in the Genetics of Lipid-Lowering drugs and diet network (GOLDN): an interventional study

**DOI:** 10.1186/1476-511X-10-181

**Published:** 2011-10-18

**Authors:** Mary K Wojczynski, Stephen P Glasser, Albert Oberman, Edmond K Kabagambe, Paul N Hopkins, Michael Y Tsai, Robert J Straka, Jose M Ordovas, Donna K Arnett

**Affiliations:** 1 Department of Biostatistics, University of Alabama at Birmingham, Birmingham, AL, USA; Current Address: Division of Statistical Genomics, Department of Genetics, Washington University in St. Louis, St. Louis, MO, USA; 2 Division of Preventive Medicine, University of Alabama at Birmingham, Birmingham, AL, USA; 3 Department of Epidemiology, University of Alabama at Birmingham, Birmingham, AL, USA; 4 Department of Cardiovascular Genetics, University of Utah, Salt Lake City, UT, USA; 5 Department of Laboratory Medicine and Pathology, University of Minnesota, Minneapolis, MN, USA; 6 Department of Experimental and Clinical Pharmacology, University of Minnesota, Minneapolis, MN, USA; 7 Department of Nutrition and Genetics, Tufts University, Boston, MA, USA

**Keywords:** postprandial lipemia, lipoprotein particles, NMR, high-fat meal

## Abstract

**Background:**

Postprandial lipemia (PPL) is likely a risk factor for cardiovascular disease but these changes have not been well described and characterized in a large cohort. We assessed acute changes in the size and concentration of total and subclasses of LDL, HDL, and VLDL particles in response to a high-fat meal. Participants (n = 1048) from the Genetics of Lipid-Lowering Drugs and Diet Network (GOLDN) Study who ingested a high-fat meal were included in this analysis. Lipids were measured at 0 hr (fasting), 3.5 hr, and 6 hr after a standardized fat meal. Particle size distributions were determined using nuclear magnetic resonance spectroscopy. Analyses were stratified by baseline triglycerides (normal vs. elevated) and gender. The effect of PPL on changes in lipoprotein subclasses was assessed using repeated measures ANOVA.

**Results:**

Postprandially, LDL-C, HDL-C, VLDL-C, and triglycerides increased regardless of baseline triglyceride status, with the largest increases in VLDL-C and TG; however, those with elevated triglycerides demonstrated larger magnitude of response. Total LDL particle number decreased over the 6-hour time interval, mostly from a decrease in the number of small LDL particles. Similarly, total VLDL particle number decreased due to reductions in medium and small VLDL particles. Large VLDL particles and chylomicrons demonstrated the largest increase in concentration. HDL particles demonstrated minimal overall changes in total particle number.

**Conclusions:**

We have characterized the changes in LDL and VLDL particle number, and their subclass patterns following a high-fat meal.

## Background

Most studies use the measurement of fasting lipoproteins to assess cardiovascular disease (CVD) risk [1,2]; however, humans spend most of the day in a postprandial state. Postprandial lipemia (PPL) is a physiological response occurring 2 to 12 hours after consuming a meal [3]. Gross lipid profile changes during PPL include increases in triglyceride (TG), very low-density lipoprotein cholesterol (VLDL-C), and chylomicron concentrations; decreases in high-density lipoprotein cholesterol (HDL-C) concentration; and little to no change in low-density lipoprotein cholesterol (LDL-C) concentration. Each of these lipoprotein classes is, however, heterogeneous and composed of various subclasses [4-6]. Lipoprotein particles are composed of different proportions of cholesterol ester (CE) and TG, and each subclass also has specific apolipoproteins on its surface for recognition of receptors and enzymes [7]. Constituent fractions of the lipoprotein profile can vary with respect to particle size, particle number, and particle concentrations [8]. The ingestion of a meal high in fat leads to distributional shifts of VLDL, LDL (including intermediate density lipoproteins (IDL)), and HDL particle sizes, numbers, and plasma concentrations [9]. The various particle sizes have also demonstrated both pro-atherogenic (large VLDL and small LDL) and anti-atherogenic (large LDL) effects [10]. Although much research examines the major lipoproteins and lipid particle sizes in relation to cardiovascular disease, only a few relatively small studies have used the interventional methodology described here to examine changes in lipoprotein distribution and subclasses in the postprandial state following a high-fat challenge [11-16].

Thus, we sought to describe in a large cohort, acute changes in lipoprotein (LDL, HDL, and VLDL) particle sizes and numbers following a high-fat meal and to determine if the lipoprotein particle changes would vary by baseline triglyceride concentrations and gender.

## Results

### Population characteristics

**Figure 1:**
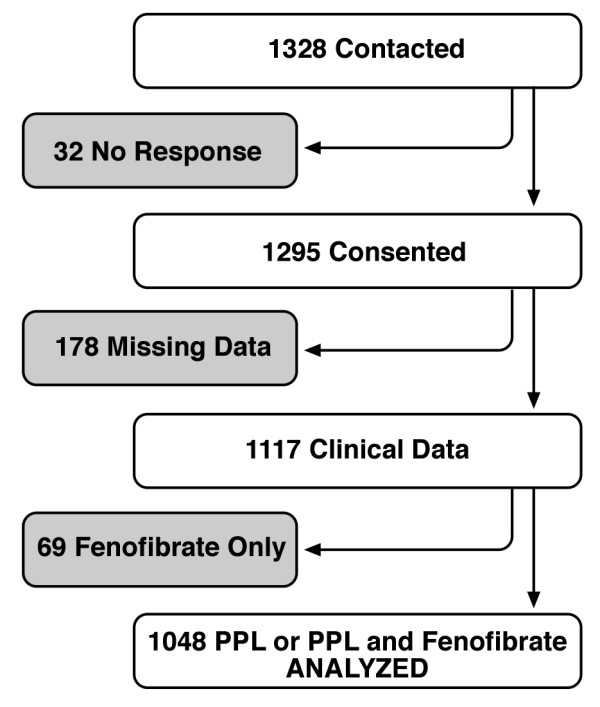
**GOLDN study recruitment**.

Table 1 shows the characteristics of the study participants according to gender and baseline triglyceride status. These 1048 subjects were part of a cohort of 1328 who were contacted, of whom 1123 participated (Figure 1). Of the 1048 subjects in the analysis, all were white, about half were female, and the mean age of the study group was 48.2 (18-87) years old (Table 1). The hyper-triglyceridemic individuals differed significantly from normal individuals in all baseline traits except smoking status.

**Table 1:** Baseline characteristics of GOLDN study population.

	Men			Women		
	
	Normal Triglyceridemic (n = 317)	Hyper-Triglyceridemic (n = 185)	p-value	Normal Triglyceridemic (n = 390)	Hyper-Triglyceridemic (n = 156)	p-value
Age, y	47.6 (17.2)	52.6 (14.5)	0.001	45.8 (16.4)	55.5 (14.7)	< 0.0001
Body mass index, kg/m^2^	27.5 (4.9)	30.1 (4.3)	< .0001	26.9 (6.0)	31.2 (6.1)	< 0.0001
Waist, cm	97.9 (16.1)	105.3 (11.5)	< .0001	89.6 (16.6)	102.0 (16.0)	< 0.0001
Hypertension, %	22.5	36.2	0.0009	19.7	36.5	< 0.0001
Diabetes, %	4.7	10.3	0.0164	5.4	18.6	< 0.0001
Insulin, mU/L	12.3 (7.0)	17.5 (9.8)	< .0001	11.7 (6.5)	18.0 (9.4)	< 0.0001
Glucose, mg/dL	101.6 (14.3)	110.6 (23.6)	< .0001	95.3 (14.3)	104.9 (18.1)	< 0.0001
Current smoker, %	6.9	8.1	0.629	7.7	5.8	0.4264
LDL-C, mg/dL	119.7 (29.3)	130.0 (29.8)	0.0002	112.8 (29.1)	137.6 (32.6)	< 0.0001
HDL-C, mg/dL	44.5 (9.5)	36.2 (7.5)	< .0001	54.4 (13.7)	46.2 (11.8)	< 0.0001
Triglycerides, mg/dL	92.9 (29.2)	244.0 (101.1)	< .0001	87.9 (31.6)	227.9 (78.0)	< 0.0001

HDL-C = high-density lipoprotein cholesterol; LDL-C = low-density lipoprotein cholesterol.

### Plasma lipid concentrations

**Figure 2:**
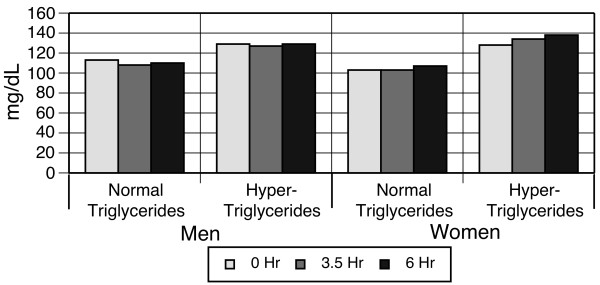
**Low-density lipoprotein cholesterol response to high-fat meal, stratified by gender and baseline triglycerides**.

Total LDL-C levels demonstrated decreases between 0 and 6 hours following the high-fat meal (113 to 110 mg/dL in the normo-triglyceridemic men; and no change among hyper-triglyceridemic men, p < 0.0001, Figure 2). Among women, LDL-C levels had small but significant (p < 0.0001) increases between 0 and 6 hours (103 to 107 mg/dL among normo-triglyceridemic women and 128 to 138 mg/dL among hyper-triglyceridemic women, Figure 2).

**Figure 3:**
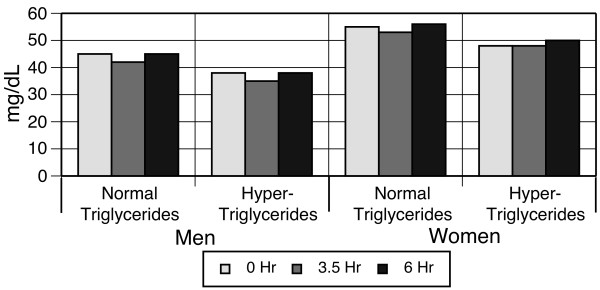
**High-density lipoprotein cholesterol response to high-fat meal, stratified by gender and baseline triglycerides**.

Among both normo- and hyper-triglyceridemic men, HDL-C levels displayed a decrease at 3.5 hours but returned to baseline levels by 6 hours, with normo-triglyceridemic men having higher HDL-C concentrations than hyper-triglyceridemic men. Normo-triglyceridemic women also had a decrease in HDL-C levels at 3.5 hours followed by a return to levels slightly above baseline at 6 hours; however, hyper-triglyceridemic women did not demonstrate a decrease at 3.5 hours and at 6 hours increased above baseline (Figure 3). Similar to men, normo-triglyceridemic women had higher HDL-C levels than hyper-triglyceridemic women. These time and triglyceridemic effects were significant (p < 0.0001).

**Figure 4:**
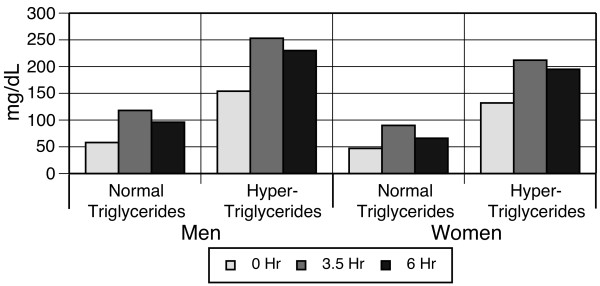
**Very low-density lipoprotein cholesterol response to high-fat meal, stratified by gender and baseline triglycerides**.

**Figure 5:**
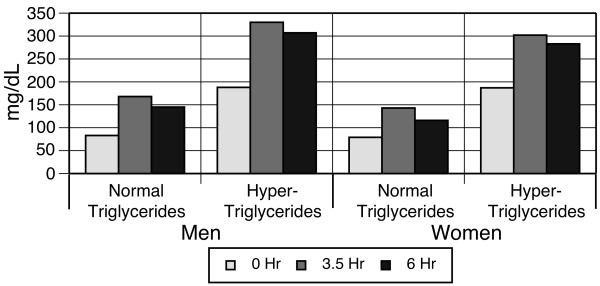
**Triglyceride response to high-fat meal, stratified by gender and baseline triglycerides**.

All groups had a similar pattern of statistically significant changes in VLDL-C levels in response to the high-fat meal: large increases at 3.5 hours were followed by a decrease at 6 hours; however, VLDL-C levels were still elevated above baseline at 6 hours (p < 0.0001). Further, the magnitude of the VLDL-C levels was higher among men than women and among hyper-triglyceridemic compared to normo-triglyceridemic individuals (Figure 4). Total triglycerides increased significantly from 83 to 145 mg/dL in the normo-triglyceridemic men and from 188 to 307 mg/dL in hyper-triglyceridemic men (p < 0.0001). Women displayed similar pattern of statistically significant changes (p < 0.0001, Figure 5).

### LDL particle size subclasses

Hyper-triglyceridemic participants had higher baseline concentrations of all LDL particle subclasses compared to normo-triglyceridemic participants (Tables 2 and 3). Additionally hyper- and normo-triglyceridemic participants displayed similar patterns of change in LDL particle size concentrations. In both the normo- and hyper-triglyceridemic men and women, there was a small but significant (p < 0.0001) decrease in total LDL particle number following the high-fat meal (normo-triglyceridemic: 1212 to 1195 nmol/L in men, and 1086 to 1076 nmol/L in women); however, the concentrations of IDL and large LDL particles demonstrated significant increases (IDL, normo-triglyceridemic: 28 to 43 nmol/L in men and 22 to 41 nmol/L in women, p < 0.0001; large LDL, normo-triglyceridemic: 406 to 452 nmol/L in men and 457 to 527 nmol/L in women, p < 0.0001), and the concentration of small LDL particles demonstrated a significant decrease (normo-triglyceridemic: 787 to 703 nmol/L in men and 611 to 518 nmol/L in women, p < 0.0001). This large decrease in small LDL particle concentration is responsible for the overall decrease in total LDL particle number (p < 0.0001). Despite significant (p < 0.0001) PPL changes, LDL particle size did not demonstrate substantial changes.

**Table 2:** Mean NMR lipoprotein particle size and number in men stratified by baseline triglyceride status.

	Normal Triglycerides			Hyper-Triglyceridemic			p-values		
	
	0 Hr	3.5 Hr	6 Hr	0 Hr	3.5 Hr	6 Hr	Triglyceride Status	Time	Triglyceride status × time
LDL particle No., nmol/L	1213 (22)	1187 (22)	1196 (22)	1609 (26)	1592 (26)	1601 (27)	< 0.0001	< 0.0001	0.5231
IDL particles, nmol/L	28 (2)	44 (3)	43 (3)	56 (3)	84 (4)	75 (3)	< 0.0001	< 0.0001	0.0002
Large LDL particles, nmol/L	406 (11)	421 (12)	452 (12)	266 (14)	308 (14)	329 (14)	< 0.0001	< 0.0001	0.0011
Small LDL particles, nmol/L	788 (21)	726 (21)	704 (22)	1268 (26)	1171 (26)	1175 (27)	< 0.0001	< 0.0001	0.0168
LDL particle size, nm	21 (0.03)	21 (0.03)	21 (0.03)	20 (0.04)	20 (0.04)	20 (0.04)	< 0.0001	< 0.0001	0.0012
HDL particle No., μmol/L	30 (0.3)	30 (0.3)	31 (0.3)	29 (0.4)	29 (0.3)	30 (0.3)	0.0034	< 0.0001	0.0157
Large HDL particles, μmol/L	5 (0.1)	5 (0.1)	5 (0.2)	3 (0.2)	3 (0.2)	3 (0.2)	< 0.0001	< 0.0001	0.0001
Medium HDL particles, μmol/L	3 (0.2)	5 (0.2)	7 (0.2)	2 (0.2)	4 (0.3)	5 (0.3)	< 0.0001	< 0.0001	0.0579
Small HDL particles, μmol/L	21 (0.3)	19 (0.3)	18 (0.3)	24 (0.4)	22 (0.4)	21 (0.4)	< 0.0001	< 0.0001	< 0.0001
HDL particle size, nm	9 (0.02)	9 (0.02)	9 (0.02)	8 (0.02)	8 (0.03)	9 (0.02)	< 0.0001	< 0.0001	< 0.0001
VLDL particle No., nmol/L	48 (2)	49 (2)	42 (2)	100 (2)	87 (2)	83 (2)	< 0.0001	< 0.0001	< 0.0001
Large VLDL particles, nmol/L	1 (0.2)	3 (0.2)	3 (0.3)	6 (0.2)	10 (0.3)	10 (0.3)	< 0.0001	< 0.0001	< 0.0001
Medium VLDL particles, nmol/L	19 (1)	28 (1)	18 (1)	55 (2)	56 (2)	48 (1)	< 0.0001	< 0.0001	< 0.0001
Small VLDL particles, nmol/L	26 (0.9)	15 (0.7)	19 (0.8)	36 (1)	18 (0.9)	24 (1)	< 0.0001	< 0.0001	< 0.0001
Chylomicrons, nmol/L	0.04 (0.01)	0.3 (0.01)	0.2 (0.02)	0.04 (0.01)	0.6 (0.02)	0.5 (0.02)	< 0.0001	< 0.0001	< 0.0001
VLDL particle size, nm	51 (0.4)	53 (0.4)	58 (0.5)	53 (0.5)	58 (0.5)	60 (0.6)	< 0.0001	< 0.0001	< 0.0001

Adjustment variables include field center, age, waist circumference, smoker, diabetes.

HDL = high-density lipoprotein; IDL = intermediate-density lipoprotein; LDL = low-density lipoprotein; NMR = nuclear magnetic resonance spectroscopy; VLDL = very low-density lipoprotein.

**Table 3:** Mean NMR lipoprotein particle size and number in women stratified by baseline triglyceride status.

	Normal Triglycerides			Hyper-Triglyceridemic			p-values		
	
	0 Hr	3.5 Hr	6 Hr	0 Hr	3.5 Hr	6 Hr	Triglyceride Status	Time	Triglyceride status × time
LDL particle No., nmol/L	1087 (19)	1061 (18)	1077 (19)	1550 (26)	1508 (26)	1531 (26)	< 0.0001	< 0.0001	0.2495
IDL particles, nmol/L	22 (2)	38 (2)	41 (2)	58 (3)	70 (3)	67 (3)	< 0.0001	< 0.0001	0.0155
Large LDL particles, nmol/L	457 (13)	486 (13)	526 (13)	426 (17)	481 (17)	522 (18)	0.1334	< 0.0001	0.0029
Small LDL particles, nmol/L	614 (20)	547 (19)	521 (19)	1054 (28)	943 (27)	926 (28)	< 0.0001	< .0001	0.0076
LDL particle size, nm	21 (0.03)	21 (0.03)	21 (0.03)	21 (0.05)	21 (0.05)	21 (0.05)	< 0.0001	< 0.0001	0.007
HDL particle No., μmol/L	33 (0.3)	32 (0.3)	32 (0.3)	35 (0.4)	34 (0.4)	35 (0.4)	0.0576	< 0.0001	0.0008
Large HDL particles, μmol/L	8 (0.2)	8 (0.2)	9 (0.2)	6 (0.3)	5 (0.3)	5 (0.3)	< 0.0001	0.0581	< 0.0001
Medium HDL particles, μmol/L	5 (0.2)	8 (0.2)	10 (0.3)	5 (0.3)	8 (0.3)	10 (0.4)	0.7465	< 0.0001	0.026
Small HDL particles, μmol/L	20 (0.3)	16 (0.3)	13 (0.3)	24 (0.4)	20 (0.4)	19 (0.5)	< 0.0001	< 0.0001	0.0002
HDL particle size, nm	9 (0.02)	9 (0.02)	9 (0.02)	9 (0.03)	9 (0.03)	9 (0.03)	< 0.0001	< 0.0001	< 0.0001
VLDL particle No., nmol/L	37 (1)	40 (1)	31 (1)	84 (2)	76 (2)	75 (2)	< 0.0001	< 0.0001	< 0.0001
Large VLDL particles, nmol/L	1 (0.2)	2 (0.2)	2 (0.2)	6 (0.2)	10 (0.3)	8 (0.3)	< 0.0001	< 0.0001	< 0.0001
Medium VLDL particles, nmol/L	15 (0.9)	20 (0.9)	11 (0.9)	39 (1)	41 (1)	36 (1)	< 0.0001	< 0.0001	< 0.0001
Small VLDL particles, nmol/L	20 (0.8)	16 (0.7)	18 (0.7)	35 (1)	23 (1)	28 (1)	< 0.0001	< 0.0001	< 0.0001
Chylomicrons, nmol/L	0.04 (0.004)	0.2 (0.01)	0.2 (0.01)	0.03 (0.005)	0.4 (0.01)	0.4 (0.02)	< 0.0001	< 0.0001	< 0.0001
VLDL particle size, nm	51 (0.4)	54 (0.4)	60 (0.5)	53 (0.6)	60 (0.6)	62 (0.8)	0.0014	< 0.0001	< 0.0001

Adjustment variables include field center, age, waist circumference, smoker, diabetes.

HDL = high-density lipoprotein; IDL = intermediate-density lipoprotein; LDL = low-density lipoprotein; NMR = nuclear magnetic resonance spectroscopy; VLDL = very low-density lipoprotein.

### HDL particle size subclasses

In response to a high-fat meal, normo- and hyper-triglyceridemic men displayed similar patterns of change in HDL particle size concentration distributions; however, the magnitudes of these patterns differed in hyper-compared to normo-triglyceridemic participants. In men, there was no effect on large HDL particle concentrations, but significant increases in the concentration of medium HDL particles (3 to 7 mmol/L in normo-triglyceridemics and 2 to 5 mmol/L in hyper-triglyceridemics, p < 0.0001) as well as significant decreases in small HDL particle concentrations (21 to 18 mmol/L in normo-triglyceridemics and 24 to 21 mmol/L in hyper-triglyceridemics, p < 0.0001). Furthermore, total HDL particle number significantly increased among men in response to the high-fat meal (p < 0.0001), which can be attributed to the large increases in medium HDL particle size concentrations.

Among both normo- and hyper-triglyceridemic individuals, women responded differently to the high-fat meal than men. Normo-triglyceridemic women demonstrated a significant decrease in total HDL particle number (p < 0.0001), while there was no overall change among hyper-triglyceridemic women in response to the meal (33 to 32 mmol/L in normo-triglyceridemic and 35 mmol/L in hyper-triglyceridemic). Additionally, these subgroups demonstrated different responses for large HDL particle concentrations where normo-triglyceridemic women displayed significant increases (8 to 9 mmol/L), and hyper-triglyceridemic women displayed significant decreases in large HDL particle concentration (6 to 5 mmol/L, p = 0.0367). For both normo- and hyper-triglyceridemic women, medium and small HDL particle concentrations demonstrated similar responses to the high-fat meal: medium HDL particle concentrations had significant increases (5 to 10 mmol/L for both subgroups, p < 0.0001), and small HDL particle concentrations had significant decreases (20 to 13 mmol/L for normo-triglyceridemics and 24 to 18 mmol/L for hyper-triglyceridemics, p < 0.0001). Similar to LDL size, no substantial change in HDL size was demonstrated in response to the high-fat meal despite its statistical significance (p < 0.0001).

### VLDL particle size subclasses

Results of the PPL VLDL particle size subclass analysis were similar in pattern for men and women, but the magnitude of change and concentration values were different, with women, on average, having lower concentrations. Total VLDL particle number decreased significantly among both normo- and hyper-triglyceridemics (among men, 48 to 42 nmol/L for normo- and 100 to 83 nmol/L for hyper-triglyceridemics, p < 0.0001). Large VLDL particle concentrations increased significantly (1 to 3 nmol/L among normo-triglyceridemic men and 6 to 10 nmol/L among hyper-triglyceridemic men, p < 0.0001). Medium and small VLDL particle concentrations demonstrated significant decreases (p < 0.0001 for both men and women). Finally, VLDL particle size significantly increased (p < 0.0001) among normo- and hyper-triglyceridemic men and women.

## Discussion

The present study focused on the PPL response to an acute (1-time) high-fat meal using data derived from the Genetics of Lipid-Lowering Drugs and Diet Network (GOLDN) Study. We sought to describe lipoprotein particle subclass number, size, and concentrations stratified by gender and baseline triglyceride status. Although the pattern of change observed for both men and women was qualitatively similar, the magnitude of these changes appeared to be more exaggerated in men compared to women, especially for VLDL and TG response. Similar to the response demonstrated by gender, the pattern of change was qualitatively similar for normo- and hyper-triglyceridemic individuals; however, the magnitude was more exaggerated among hyper-triglyceridemic than normo-triglyceridemic individuals.

Specifically, in terms of lipoprotein particles for both normo- or hyper-triglyceridemic individuals, we observed little change in total particle number over the 3 time points (up to 6 hours after the meal) for either total LDL or HDL particle number. In contrast, for normo-triglyceridemic subjects, total VLDL particle number appeared to rise above baseline at 3.5 hours and drop considerably below baseline at 6 hours after the high-fat meal. This pattern was considerably different in hyper-triglyceridemic individuals where, in both men and women, we observed a precipitous drop relative to baseline which continued from the 3.5 to the 6 hour time point.

Despite expected baseline differences between normo- and hyper-triglyceridemic individuals, there was a reduction in small LDL particle concentrations secondary to the PPL challenge. In normo-triglyceridemic individuals there was a persistent decline from baseline, while in hyper-triglyceridemic men and women small LDL particle concentration appeared to plateau.

The major pattern we observed among HDL particle subclasses was a significant shift to more medium HDL particles and fewer small HDL particles with a non-significant increase in HDL-C (p > 0.05 for men and women). Decewicz et al [9] observed decreases in medium HDL particles in a 73 case - 73 control study of a 1-year dietary lifestyle intervention; this contrasts to the observed increase in medium HDL observed among the 546 women and 502 men studied in the GOLDN cohort after an acute high-fat meal. It is likely that sample size and diet (Ornish diet low in fat [9] vs high fat (GOLDN)) are at the root of these differences in response. In a study examining the difference in particle distribution between diabetics and non-diabetics by Colhoun et al [10], diabetics had a lower concentration of small HDL and a larger concentration of large HDL and larger HDL sizes [10], a finding similar to ours; however, the Colhoun et al study was a descriptive study of fasting particle distributions and did not include a fat-loading intervention.

In our study, the total number of VLDL particles decreased during the postprandial period which was due to an increase in large VLDL and chylomicrons along with more substantial decreases in medium and small VLDL subclasses. A decrease in medium VLDL and total VLDL particles was also observed among the diabetic subjects in the study by Colhoun et al [10]. Our study contained a small proportion of diabetics, thus rendering further stratification by diabetic status unfeasible. Furthermore, decreases in small/medium VLDL particles were demonstrated among women during the therapeutic lifestyle change diet by Li et al [11]. The distributional shift in VLDL particle subclasses was consistent and somewhat expected since large VLDL and chylomicrons accept triglycerides in an effort to quickly repackage and remove them from the circulation [7]; however, the small reduction in total VLDL particles is unexpected, especially since VLDL-C increased in response to the acute high-fat meal.

During the PPL response to a high-fat meal, many changes occur during lipoprotein metabolism. It has been shown that HDL becomes enriched with TG, leading to increased removal of HDL from the plasma, thereby decreasing HDL-C [17]. Also the transfer of cholesterol ester (CE), the main component of HDL, is increased due to the increased activity of cholesteryl ester transfer protein (CETP) during PPL [18], and CE is preferably transferred to chylomicrons [17], all of which are demonstrated in our study by a postprandial decrease in HDL-C and increase in chylomicrons. Similarly CE from LDL is also transferred to chylomicrons during PPL [18]. Hepatic lipase (HL) activity also increases during PPL, thus catabolizing both HDL and LDL into smaller, denser HDL and LDL particles [17,19]; however, increases in smaller, denser HDL and LDL were not clearly demonstrated in our population after a high-fat meal. Since it is generally believed that smaller LDL particles are more atherogenic [20], further characterization and understanding of the lipoprotein fluctuations that occur due to an acute high-fat meal may enhance our understanding of the relationship of lipoprotein particle subclasses to the increased risk for CVD.

This study differs from the majority of previous studies examining dietary influences on PPL that were conducted in smaller study populations, utilized different dietary interventions, and did not report changes in LDL particle subclasses [11,12]. For example, Li et al [11] studied 33 subjects and showed that therapeutic lifestyle changes focused on diet decreased IDL among men, but not women. This is opposite to what we observed, and this is likely due to differences in the dietary intervention of Li et al [11] who utilized a diet low in total fat, saturated fat, and cholesterol, compared to the GOLDN intervention which was high in total fat and cholesterol. Additionally, random variation could explain differences since the Li et al [11] study evaluated 33 subjects compared to the 1048 reported from GOLDN.

The strengths of this study are its large sample size, the use of a standardized acute fat load, and the use of NMR technology to characterize particle size subclasses. To our knowledge, it is the largest PPL study conducted to date. Our study did not measure serum apolipoprotein B and is not a CVD outcome or mechanistic study; therefore, we do not know for certain how or why these lipoprotein particle distributional shifts occur or how they might impact CVD risk. Nonetheless, among hyper-triglyceridemic individuals, the prolonged return to baseline levels may add to an already elevated atherogenic profile [18].

Additionally, we did not obtain information on the activity level of enzymes and proteins that are known to be activated during the PPL response, such as CETP and HL [18]. Specific knowledge of how these enzymes and proteins respond during an acute high-fat meal challenge would greatly enhance the understanding of our findings as well as further the understanding of lipoprotein metabolism. We only examined the acute response to 1 high-fat meal, but since humans are in a non-fasting postprandial state for a majority of the day [21], the changes that we demonstrated may actually never return to baseline except during the latter phases of sleep. Thus, it may be the length of time that these changes in lipoprotein particle fluctuations exist that is important in atherogenesis. Another possible mechanism for atherogenesis of the particle subclasses is the fluctuations in the TG:CE content of the particles [18]. Since each lipoprotein particle subclass is composed of varying proportions of TG and CE, it may be that the changes in those proportions are atherogenic. Lastly, these findings may not extend to other racial groups, as our study population was all white.

## Conclusions

Postprandial lipemia persists for at least 6 hours following a high-fat meal. We confirmed that men differ from women in the PPL response, and that the PPL response varies based on baseline triglyceride profile. We demonstrated an increase in IDL particles, especially in normo-triglyceridemic women, and an increase in triglycerides in both men and women. Large HDL also changed in hyper-triglyceridemic women (17% decrease was observed), which is interesting in view of the clinically observed greater risk of CVD in hyper-triglyceridemic women compared to men [22]. Overall, VLDL particles decreased but triglyceride-rich large VLDL and chylomicrons (with their propensity to generate atherogenic remnants) increased dramatically; however, the actual magnitude of change in total VLDL particles was relatively small. As our results demonstrate, the lipoprotein fluctuations are distinct, and some are not intuitive based on our existing knowledge of lipids and lipoprotein metabolism. Finally, our findings provide further mechanistic insight into the changes in lipoprotein metabolism occurring after an acute high-fat meal.

## Methods

### Study population

The Genetics of Lipid-Lowering Drugs and Diet Network (GOLDN) study population consisted of 189 families who were recruited from 3-generational pedigrees from the NHLBI Family Heart Study Minneapolis, MN, and Salt Lake City, UT, field centers. The overarching intent of GOLDN was to identify loci contributing to phenotypic variation in response to a high-fat meal and fenofibrate therapy. The Institutional Review Boards at the University of Alabama, the University of Minnesota, the University of Utah, and Tufts University approved the study protocol.

### Inclusion and exclusion criteria

Participants had to meet the following criteria: White (other racial groups were excluded by design since the recruitment sources were extant cohorts of white families from Utah and Minnesota), male or female, ≥ 18 years of age, fasting triglycerides (TGs) < 1500 mg/dL, willingness to participate in the study and attend the scheduled clinic exams, part of a family with at least 2 members in a sibship, AST and ALT tests within normal range, and creatinine ≤ 2.0. Exclusion criteria for the fenofibrate intervention included the following: history of liver, kidney, pancreas, or gall bladder disease or malabsorption; pregnancy or women of childbearing potential not using an acceptable form of contraception; insulin use; known hypersensitivity to fenofibrate; pancreatitis within 12 months prior to enrollment; and current use of warfarin and/or nutraceuticals. Subjects using hypolipidemic drugs were asked to consult with their physicians to determine whether they could be taken off medications for 4 weeks prior to and for the duration of the study; informed consent for drug withdrawal was obtained. Only subjects who remained untreated with any prescription or over-the-counter hypolipidemic medication were eligible.

### Study design

Participants underwent 2 interventions: an acute fat loading dietary challenge and a short-term (3 weeks) open-label intervention trial of fenofibrate. The specific methodology of GOLDN is reported elsewhere [23,24]. For this analysis, we report LDL, HDL, and VLDL particle subclass concentrations, size and number in response to an acute standardized high fat challenge.

The high-fat challenge followed the protocol of Patsch et al [25]. The caloric intake of the intervention meal was determined by body surface area, containing 700 kilocalories per m^2 ^of body surface area (2.93 MJ/m^2 ^body surface area). The meal composition was 83% of calories from fat, 14% from carbohydrates, and 3% from protein. The meal was formulated to have a cholesterol content of 240 mg and a polyunsaturated:saturated fat ratio of 0.06. Based on these guidelines, the average individual ingested 175 mL of heavy whipping cream (39.5% fat) combined with 7.5 mL powdered, instant, non-fat, dry milk, and blended with ice. To increase palatability of the drink, 15 mL of chocolate- or strawberry-flavored syrup was also added. Participants had 15 minutes to ingest this meal and were required to fast a minimum of 8 hours prior to the meal. Immediately before ingestion, we drew blood samples on all participants (0 hr, fasting), and then again at 3.5 and 6 hours after the high-fat meal.

### Biochemical assays

Serum and EDTA-anticoagulant tubes were collected and processed using a standardized protocol, aliquoted, and stored at -70°C until time of use. Analysis was completed on all stored samples at the end of the study and all samples for an individual were processed in the same batch to reduce measurement error. Triglyceride measurements were calculated using the glycerol-blanked enzymatic method on the Roche COBAS FARA centrifugal analyzer (Roche Diagnostics Corporation). Hyper-triglyceridemia was defined as a baseline triglyceride ≥ 150 mg/dL. HDL-C was calculated using the same procedure as TG measurement after precipitation of non-HDL cholesterol with magnesium/dextran. LDL-C measurement employed a homogeneous direct method (LDL Direct Liquid Select™ Cholesterol Reagent; Equal Diagnostics) on a Hitachi 911 Automatic Analyzer.

### Lipoprotein subclass analysis

**Figure 6:**
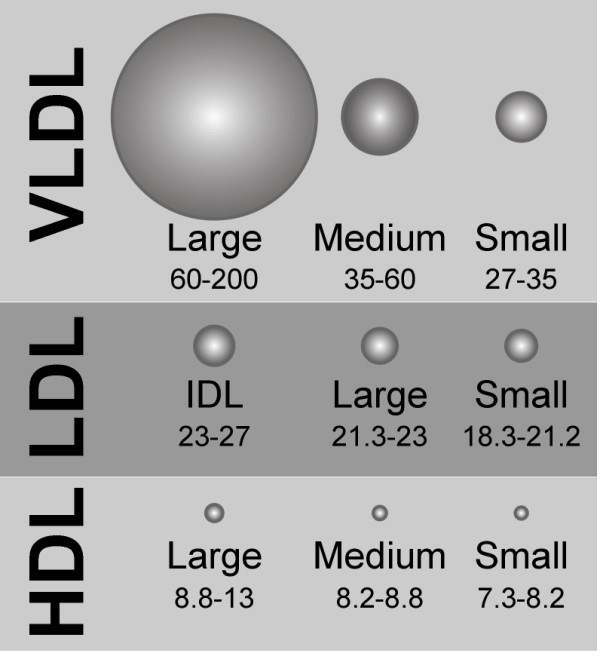
**Nuclear magnetic resonance-derived low-density lipoprotein (LDL), high-density lipoprotein (HDL), and very low-density lipoprotein (VLDL) particle size subclasses**.

Proton nuclear magnetic resonance (NMR) spectroscopy was used to determine LDL, HDL, and VLDL particle size subclass concentrations (Liposcience, Raleigh, NC). Measured amplitudes after exposure to a 400 MHz magnet and known distinct lipid methyl group NMR spectroscopic profiles were used to determine the subclass concentrations [8,26-30]. Concentrations of LDL and VLDL subclasses are reported as nanomoles per liter and HDL is reported as mmol/L. The different lipoprotein particles correspond to the following average size in nanometers (nm): intermediate density lipoprotein (IDL), 23-27 nm; large LDL, 21.2-23 nm; small LDL, 18-21.2 nm; large HDL, 8.8-13 nm; medium HDL, 8.2-8.8 nm; small HDL, 7.3-8.2 nm; large VLDL, 60-200 nm; medium VLDL, 35-60 nm; and small VLDL, 27-35 nm [8,11,31]; relative size is depicted in Figure 6.

### Statistical methods

All analyses were conducted separately for men and women and stratified by baseline hyper-versus normo-triglyceride levels (TG > 150 vs TG ≤ 150) due to significant interactions between gender and baseline triglyceride concentrations in analyses for LDL, HDL, and VLDL. To determine the significance of differences in percentages between male and female normo- and hyper-triglyceridemic individuals, we used the Pearson c^2 ^and Fisher exact tests. To compare crude means, we used ANOVA and Student's t-test. Results are expressed as least squares mean (standard error). We examined changes in subclass particle size concentrations that occurred during the dietary fat challenge (baseline, 3.5 hours, and 6 hours) using repeated-measures ANOVA in which the family was modeled as a random variable. All statistical calculations were performed using SAS 9.1 (SAS Institute, Inc.).

## List of abbreviations used

ALT: alanine transaminase; AST: aspartate transaminase; CETP: cholesteryl ester transfer protein; CVD: cardiovascular disease; GOLDN: Genetics of Lipid-Lowering Drugs and Diet Network Study; HDL-C: high-density lipoprotein cholesterol; HL: hepatic lipase; IDL-C: intermediate-density lipoprotein; LDL-C: low-density lipoprotein cholesterol; NMR: nuclear magnetic resonance (spectroscopy); PPL: post-prandial lipemia; TG: triglyceride; VLDL-C: very low-density lipoprotein cholesterol

## Competing interests

The authors declare that they have no competing interests.

## Authors' contributions

MKW conducted the statistical analyses and drafted the manuscript. AO and EKK provided critical input within their respective areas of expertise during analysis, drafting, review, and revision. SPG, PNH, MYT, RJS, and JMO were critical participants in study design and provided input within their respective areas of expertise during analysis, drafting, review, and revision. DKA was the primary designer of the study and oversaw and contributed to all stages of analysis and authoring. All authors read and approved the final manuscript.

## References

[B1] Expert Panel on Detection Treatment of High Blood Cholesterol in Adults Executive Summary of The Third Report of The National Cholesterol Education Program (NCEP) Expert Panel on Detection, Evaluation, And Treatment of High Blood Cholesterol In Adults (Adult Treatment Panel III) JAMA 2001 285 2486 2497 10.1001/jama.285.19.2486 11368702

[B2] Wilson PW D'Agostino RB Levy D Belanger AM Silbershatz H Kannel WB Prediction of coronary heart disease using risk factor categories Circulation 1998 97 1837 1847 9603539 10.1161/01.cir.97.18.1837

[B3] Roche HM Gibney MJ The impact of postprandial lipemia in accelerating atherothrombosis J Cardiovasc Risk 2000 7 317 324 11143761 10.1177/204748730000700504

[B4] Cromwell WC Otvos JD Low-density lipoprotein particle number and risk for cardiovascular disease Curr Atheroscler Rep 2004 6 381 387 10.1007/s11883-004-0050-5 15296705

[B5] Otvos JD Jeyarajah EJ Cromwell WC Measurement issues related to lipoprotein heterogeneity Am J Cardiol 2002 90 22i 29i 12419478 10.1016/s0002-9149(02)02632-2

[B6] Mudd JO Borlaug BA Johnston PV Kral BG Rouf R Blumenthal RS Kwiterovich PO Jr Beyond low-density lipoprotein cholesterol: defining the role of low-density lipoprotein heterogeneity in coronary artery disease J Am Coll Cardiol 2007 50 1735 1741 10.1016/j.jacc.2007.07.045 17964036

[B7] Karpe F Postprandial lipoprotein metabolism and atherosclerosis J Intern Med 1999 246 341 355 10.1046/j.1365-2796.1999.00548.x 10583705

[B8] Otvos JD Measurement of lipoprotein subclass profiles by nuclear magnetic resonance spectroscopy Clin Lab 2002 48 171 180 11934219

[B9] Decewicz DJ Neatrour DM Burke A Haberkorn MJ Patney HL Vernalis MN Ellsworth DL Effects of cardiovascular lifestyle change on lipoprotein subclass profiles defined by nuclear magnetic resonance spectroscopy Lipids Health Dis 2009 8 26 10.1186/1476-511X-8-26 19563671 PMC2713234

[B10] Colhoun HM Otvos JD Rubens MB Taskinen MR Underwood SR Fuller JH Lipoprotein subclasses and particle sizes and their relationship with coronary artery calcification in men and women with and without type 1 diabetes Diabetes 2002 51 1949 1956 10.2337/diabetes.51.6.1949 12031985

[B11] Li Z Otvos JD Lamon-Fava S Carrasco WV Lichtenstein AH McNamara JR Ordovas JM Schaefer EJ Men and women differ in lipoprotein response to dietary saturated fat and cholesterol restriction J Nutr 2003 133 3428 3433 14608054 10.1093/jn/133.11.3428

[B12] James RW Pometta D Postprandial lipemia differentially influences high density lipoprotein subpopulations LpAI and LpAI, AII J Lipid Res 1994 35 1583 1591 7806972

[B13] Chen YD Swami S Skowronski R Coulston A Reaven GM Differences in postprandial lipemia between patients with normal glucose tolerance and noninsulin-dependent diabetes mellitus J Clin Endocrinol Metab 1993 76 172 177 10.1210/jc.76.1.172 8421086

[B14] Groot PH van Stiphout WA Krauss XH Jansen H van Tol A van Ramshorst E Chin-On S Hofman A Cresswell SR Havekes L Postprandial lipoprotein metabolism in normolipidemic men with and without coronary artery disease Arterioscler Thromb 1991 11 653 662 10.1161/01.ATV.11.3.653 2029503

[B15] Syvanne M Talmud PJ Humphries SE Fisher RM Rosseneu M Hilden H Taskinen MR Determinants of postprandial lipemia in men with coronary artery disease and low levels of HDL cholesterol J Lipid Res 1997 38 1463 1472 9254071

[B16] Couillard C Bergeron N Prud'homme D Bergeron J Tremblay A Bouchard C Mauriege P Despres JP Gender difference in postprandial lipemia: importance of visceral adipose tissue accumulation Arterioscler Thromb Vasc Biol 1999 19 2448 2455 10.1161/01.ATV.19.10.2448 10521375

[B17] Cohn JS Postprandial lipemia and remnant lipoproteins Clin Lab Med 2006 26 773 786 10.1016/j.cll.2006.07.003 17110239

[B18] Chung BH Liang P Doran S Cho BH Franklin F Postprandial chylomicrons: potent vehicles for transporting cholesterol from endogenous LDL+HDL and cell membranes to the liver via LCAT and CETP J Lipid Res 2004 45 1242 1255 10.1194/jlr.M300350-JLR200 15102891

[B19] Berneis KK Krauss RM Metabolic origins and clinical significance of LDL heterogeneity J Lipid Res 2002 43 1363 1379 10.1194/jlr.R200004-JLR200 12235168

[B20] Cromwell WC Otvos JD Keyes MJ Pencina MJ Sullivan L Vasan RS Wilson PW D'Agostino RB LDL Particle Number and Risk of Future Cardiovascular Disease in the Framingham Offspring Study - Implications for LDL Management J Clin Lipidol 2007 1 583 592 10.1016/j.jacl.2007.10.001 19657464 PMC2720529

[B21] Lopez-Miranda J Williams C Lairon D Dietary, physiological, genetic and pathological influences on postprandial lipid metabolism Br J Nutr 2007 98 458 473 10.1017/S000711450774268X 17705891

[B22] Hokanson JE Austin MA Plasma triglyceride level is a risk factor for cardiovascular disease independent of high-density lipoprotein cholesterol level: a meta-analysis of population-based prospective studies J Cardiovasc Risk 1996 3 213 219 10.1097/00043798-199604000-00014 8836866

[B23] Corella D Arnett DK Tsai MY Kabagambe EK Peacock JM Hixson JE Straka RJ Province M Lai CQ Parnell LD Borecki I Ordovas JM The -256T > C polymorphism in the apolipoprotein A-II gene promoter is associated with body mass index and food intake in the genetics of lipid lowering drugs and diet network study Clin Chem 2007 53 1144 1152 10.1373/clinchem.2006.084863 17446329

[B24] Lai CQ Arnett DK Corella D Straka RJ Tsai MY Peacock JM Adiconis X Parnell LD Hixson JE Province MA Ordovas JM Fenofibrate effect on triglyceride and postprandial response of apolipoprotein A5 variants: the GOLDN study Arterioscler Thromb Vasc Biol 2007 27 1417 1425 10.1161/ATVBAHA.107.140103 17431185

[B25] Patsch JR Miesenbock G Hopferwieser T Muhlberger V Knapp E Dunn JK Gotto AM Jr Patsch W Relation of triglyceride metabolism and coronary artery disease. Studies in the postprandial state Arterioscler Thromb 1992 12 1336 1345 10.1161/01.ATV.12.11.1336 1420093

[B26] Ordovas JM Cupples LA Corella D Otvos JD Osgood D Martinez A Lahoz C Coltell O Wilson PW Schaefer EJ Association of cholesteryl ester transfer protein-TaqIB polymorphism with variations in lipoprotein subclasses and coronary heart disease risk: the Framingham study Arterioscler Thromb Vasc Biol 2000 20 1323 1329 10.1161/01.ATV.20.5.1323 10807749

[B27] Tsai MY Georgopoulos A Otvos JD Ordovas JM Hanson NQ Peacock JM Arnett DK Comparison of ultracentrifugation and nuclear magnetic resonance spectroscopy in the quantification of triglyceride-rich lipoproteins after an oral fat load Clin Chem 2004 50 1201 1204 10.1373/clinchem.2004.032938 15142979

[B28] Otvos JD Jeyarajah EJ Bennett DW Krauss RM Development of a proton nuclear magnetic resonance spectroscopic method for determining plasma lipoprotein concentrations and subspecies distributions from a single, rapid measurement Clin Chem 1992 38 1632 1638 1326420

[B29] Staels B Vu-Dac N Kosykh VA Saladin R Fruchart JC Dallongeville J Auwerx J Fibrates downregulate apolipoprotein C-III expression independent of induction of peroxisomal acyl coenzyme A oxidase. A potential mechanism for the hypolipidemic action of fibrates J Clin Invest 1995 95 705 712 10.1172/JCI117717 7860752 PMC295538

[B30] Staels B Dallongeville J Auwerx J Schoonjans K Leitersdorf E Fruchart JC Mechanism of action of fibrates on lipid and lipoprotein metabolism Circulation 1998 98 2088 2093 9808609 10.1161/01.cir.98.19.2088

[B31] Ikewaki K Tohyama J Nakata Y Wakikawa T Kido T Mochizuki S Fenofibrate effectively reduces remnants, and small dense LDL, and increases HDL particle number in hypertriglyceridemic men - a nuclear magnetic resonance study J Atheroscler Thromb 2004 11 278 285 10.5551/jat.11.278 15557710

